# Neuropsychological measures of attention and memory function in schizophrenia: relationships with symptom dimensions and serum monoamine activity

**DOI:** 10.1186/1744-9081-1-14

**Published:** 2005-08-09

**Authors:** Robert D Oades, Bernd Röpcke, Uwe Henning, Ansgard Klimke

**Affiliations:** 1 Biopsychology Research Group, University Clinic for Child and Adolescent Psychiatry, Virchowstr. 174, 45147 ESSEN, Germany; 2 University Clinic for Psychiatry and Psychotherapy, Bergische Landstr. 2,40629 Düsseldorf, Germany

**Keywords:** schizophrenia, cognition, disorganised, ideas of reference, paranoid, dopamine, noradrenaline, serotonin

## Abstract

**Background:**

Some clinical symptoms or cognitive functions have been related to the overall state of monoamine activity in patients with schizophrenia, (e.g. inverse correlation of the dopamine metabolite HVA with delusions or visual-masking performance). However, profiles (as presented here) of the relations of the activity of dopamine, noradrenaline and serotonin to neuropsychologic (dys)functions in major patient sub-groups with their very different symptomatic and cognitive characteristics have not been reported.

**Methods:**

Serum measures of dopamine, noradrenaline and serotonin turnover were examined by regression analyses for the prediction of performance on 10 neuropsychological measures reflecting left- and right-hemispheric and frontal-, parietal- and temporal-lobe function in 108 patients with schizophrenia and 63 matched controls. The neuropsychological battery included tests of verbal fluency, Stroop interference, trail-making, block-design, Mooney faces recognition, picture-completion, immediate and delayed visual and verbal recall. Paranoid and nonparanoid subgroups were based on ratings from the Positive and Negative Syndrome Scale (PANSS). Groups with high and low ratings of ideas-of-reference and thought-disorder were formed from a median split on the Scale for Assessment of Positive Symptoms (SAPS).

**Results:**

Verbal-fluency and Stroop-interference (left frontal and fronto-cingulate function) were negatively associated with noradrenergic turnover in nonparanoid and thought-disordered patients. High dopamine turnover related to speeded trail-making (frontal modulation of set switching) in those with many ideas-of-reference. In contrast, low dopamine turnover predicted poor recall in nonparanoid patients and those with little thought disorder. Serotonin metabolism did not independently contribute to the prediction any measure of cognitive performance. But, with regard to the relative activity between monoaminergic systems, increased HVA/5-HIAA ratios predicted visual-reproduction and Mooney's face-recognition performance (right-hemisphere functions) in highly symptomatic patients. Decreased HVA/MHPG predicted non-verbal recall.

**Conclusion:**

Clinical state and function are differentially sensitive to overall levels of monoamine activity. In particular, right-lateralised cerebral function was sensitive to the relative activities of the monoamines. Increased noradrenergic activity was associated with enhanced frontal but impaired temporal lobe function in nonparanoid syndromes. Low dopaminergic activity predicted poor attentional set control in those with ideas-of-reference, but poor recall in nonparanoid patients. These data, especially the HVA/5-HIAA ratios, provide a basis for planning the nature of antipsychotic treatment aimed at patient specific symptom dimensions and cognitive abilities.

## Background

The idea that there may be a "*group *of schizophrenias" among the psychotically ill is largely attributable to Bleuler [1]. Yet, only in the last decade or so have studies of the biological concomitants of schizophrenia explicitly studied the 3–4 symptom dimensions making up the syndrome and taken account of their variation across patient samples or within individuals over time. Carpenter et al. noted that "Studies comparing a schizophrenia group with a comparison group often show differences without clarifying whether the difference relates only to a subgroup of the subjects" [2]. The reverse also occurs. Many studies report there is no difference between subject groups without considering the potentially opposing influences in the constituent subgroups (e.g. paranoid *vs. *nonparanoid schizophrenia, [3]).

Few clinicians would expect measures of attentive abilities in thought-disorganised patients to resemble those from patients with nonparanoid negative symptoms, yet few report on the biological variations that might underlie, contribute to or modulate these differences. Here we report on some indicators of such modulation (monoamine transmitters) and the association of their activity with measures of cognitive abilities in subgroups of schizophrenia.

Such contrasts are illustrated in reviews by Amin et al.[4,5]. Negative relations of the dopamine (DA) metabolite homovanillic acid (HVA) with numerous negative symptoms and positive relationships with productive symptoms have been reported frequently. Yet for *summed *ratings of negative symptoms [6] no correlations with plasma levels of HVA have been reported [7-9]. Just occasionally, when a general diagnosis of schizophrenia has been used, some biochemical associations with groups of symptoms have been described (e.g. HVA correlated positively with anhedonia-asociality ratings [9], or negatively with depression and hostility [10]).

Peripheral measures of monoamine metabolism may be weak indicators of activity affecting the central systems: but the changes that are registered are strong enough to be relevant to the study of behaviour [12]. In particular, the ratio of DA to serotonin (5-HT) metabolites (HVA/5-HIAA: [5-hydroxyindole-acetic acid]) characterises some patient groups. Low ratios are evident in thought-disordered patients but high ratios in those with prominent ideas-of-reference [11]. The amelioration of (usually) positive symptoms parallels changes in plasma HVA [13,14] and these changes can be reflected in both plasma and cerebrospinal fluid (CSF) samples [15-17]. For CSF, similar correlations for the HVA/5-HIAA ratio with both symptoms and cognitive performance have been reported [18,19]. Low CSF HVA/5-HIAA ratios were associated with a persistent impairment and a poor outcome [20], and increased ratios with a good response to treatment [21]. As would be predicted from these CSF results, plasma HVA/5-HIAA ratios rose in responders to treatment with antipsychotic drugs [22] and approached normal levels after atypical *vs*. typical neuroleptic medication [11]. Thus, as extensively argued before [4,5], plasma/serum measures of monoamine metabolism and especially the ratio between their metabolites serve as a useful proxy for widespread and general changes associated with illness and medication.

There are several findings from studies of cognitive performance that parallel the symptom-transmitter relationships. Catecholamine metabolism affects early automatic information processing in schizophrenia. Thus, DA metabolism modulates the duration of the masking influence in backward-masking tasks [23], and sensory gating of the startle response and event-related potentials [24,25]. The improvement of gating was associated with reductions of DA turnover (HVA/DA, [26,27]) and the noradrenaline (NA) metabolite (MHPG, [28]). Subsequent controlled information processing can also be influenced by each of the monoamines. Low levels of CSF HVA (but not other metabolites) correlate with poor executive functions, such as card-sorting and visuo-spatial recall [29]. Csernansky et al. [30] reported that CSF levels of HVA correlated with those of 5-HIAA, but only the latter related to performance on WAIS tests reflecting attention-related abilities (digit-span, digit-symbol, picture arrangement). With serum measures increased 5-HT turnover predicted not only the better gating of evoked potentials, but improved Tower-of-London executive function, especially for patients with symptoms of disorganisation [26].

In this study the cognitive tasks were chosen to reflect attentional and memory functions of the frontal and temporal lobes frequently impaired in schizophrenia [31]. We have reported on the neuropsychological performance and on the characterisation of subgroups in terms of monoamine metabolism: [11,32,33]. These are summarised in the appropriate sections. Here we look at the associations of monoamine activity with neuropsychological abilities, and whether such associations relate to major symptom dimensions (e.g. paranoid, thought-disorder and ideas-of-reference). The aim is to improve understanding of the bases modulating cognition and the expression of symptoms in patients with schizophrenia and with such a profile to improve the monitoring and titration of therapeutic measures for patients with differing symptom dimensions.

Based on the results reviewed above, we hypothesise 1) that some features apparently characteristic of the whole patient group (e.g. catecholamine activity) would be largely attributable to a given sub-group (e.g. paranoid *vs. *nonparanoid), with consequences for these patients' neuropsychological abilities and antipsychotic drug responses. More specifically, we suggest 2) that ratios of DA to 5-HT activity (e.g. HVA/5-HIAA) would predict cognitive impairments in disorganised patients, and that 3) ideas-of-reference, (rarely the target of investigation but with some similarities to productive symptoms) would be associated with catecholamine activity. It is important to bear in mind that the results reflect interactions of the type of illness and treatment and do not reflect exclusively the one or the other. Thus we discuss the results (i.e. the relationship of monoamine activity to task performance in subgroups of patients) separately from three perspectives, – profiles for symptom dimensions, for neuropsychological function and for monoaminergic activity, in turn.

## Results

### Clinical Groups

Patients had been ill on average for 9.5y after onset of the disorder at 23.6y and a first admission at 25.2y. The groups did not differ in age, socio-economic status of the parents or years in education. But the patients did show a lower non-verbal IQ (*F*_1,161 _= 49.4, *P *< 0.0001: table 1).

**Table 1:** Demographic and clinical data (means ± standard deviation) for 101 patients and 63 controls providing biochemical data on the left and neuropsychological performance scores on the right.

	Demographic & Clinical Data		Neuropsychological Tasks & Performance		
	Schizophrenics	Controls	Task	Schizophrenics	Controls
Age (years)	33.6 (11.0)	32.8 (11.0)	Verbal fluency	28.6 (10.8)	35.0 (9.3)
Gender (m/f)	68 / 40	34 / 29	Mooney faces (hits)	9.8 (5.7)	8.6 (4.3)
Socio-economic group^1^	4.5 (1.9)	4.9 (1.6)	Picture completion	11.8 (2.5)	14.3 (2.5)
Education (years)	13.3 (3.8)	13.7 (3.3)	Block design	24.1 (9.5)	31.4 (7.7)
IQ (short APM)	6.9 (2.9)	9.9 (1.9)	Vis Reprod (VR)	31.1 (5.0)	38.0 (3.5)
Handedness (Edinburgh)	17.5 (8.5)	18.9 (5.3)	VR + delay	24.1 (9.1)	34.8 (6.0)
Onset-Age (years)	23.6 (8.4)		Prose Reprod (PR)	17.7 (6.9)	29.8 (6.7)
First admission (years)	25.1 (9.6)		PR + delay	13.1 (6.8)	26.3 (6.3)
Duration of illness (years)	9.5 (8.0)		Trails B-A (sec)	79 (56)	35 (20)
Episode duration (days)	44.0 (40.3)		Stroop Interfere (sec)	112 (38)	80 (20)
Diagnosis-					
paranoid	70				
disorganised	24				
catatonic/residual	7				
Symptoms,					
PANSS positive	16.3 (6.0)				
negative	18.6 (8.1)				
general	36.8 (9.3)				
SAPS ideas/reference	3.2 (3.8)				
thought disorder	8.4 (6.5)				
Extrapyramidal symptoms	5.8 (5.6)				
AIMS	8.4 (2.9)				
Antipsychotic drug dose					
(CPZ: n = 99)^2^	665 (328)				
typical + risperidone	572 (n = 49, 63% male, incl. 53% of paranoid, 29% of nonparanoid groups)				
clozapine/olanzapine/sertindole	718 (n = 43, 67% male, incl. 33% of paranoid, 58% of nonparanoid groups)				
Both	678 (n = 15, 47% male, incl. 14% of paranoid, 13% of nonparanoid groups)				
Biperidene(mg/day: n = 15)	4.2 (1.8)				

**1. **Scale 1–7, [84]: AIMS, Abnormal involuntary movement scale (n = 68/108 and 41/72); APM, Advanced progressive matrices; **2. **CPZ, chlorpromazine equivalents, excluding 2 patients without medication; Biperidene is an anticholinergic drug (n = 15/108 and 9/72); PANSS, Positive and negative syndrome scale; SAPS, scale for assessment of positive symptoms; all neuropsychological tasks showed a significant group difference (p < 0.01) on point scores (low for patients) or latency (high for patients) except the Mooney faces test.

### Group and Sub-Group Biochemical Measures

**Figure 1:**
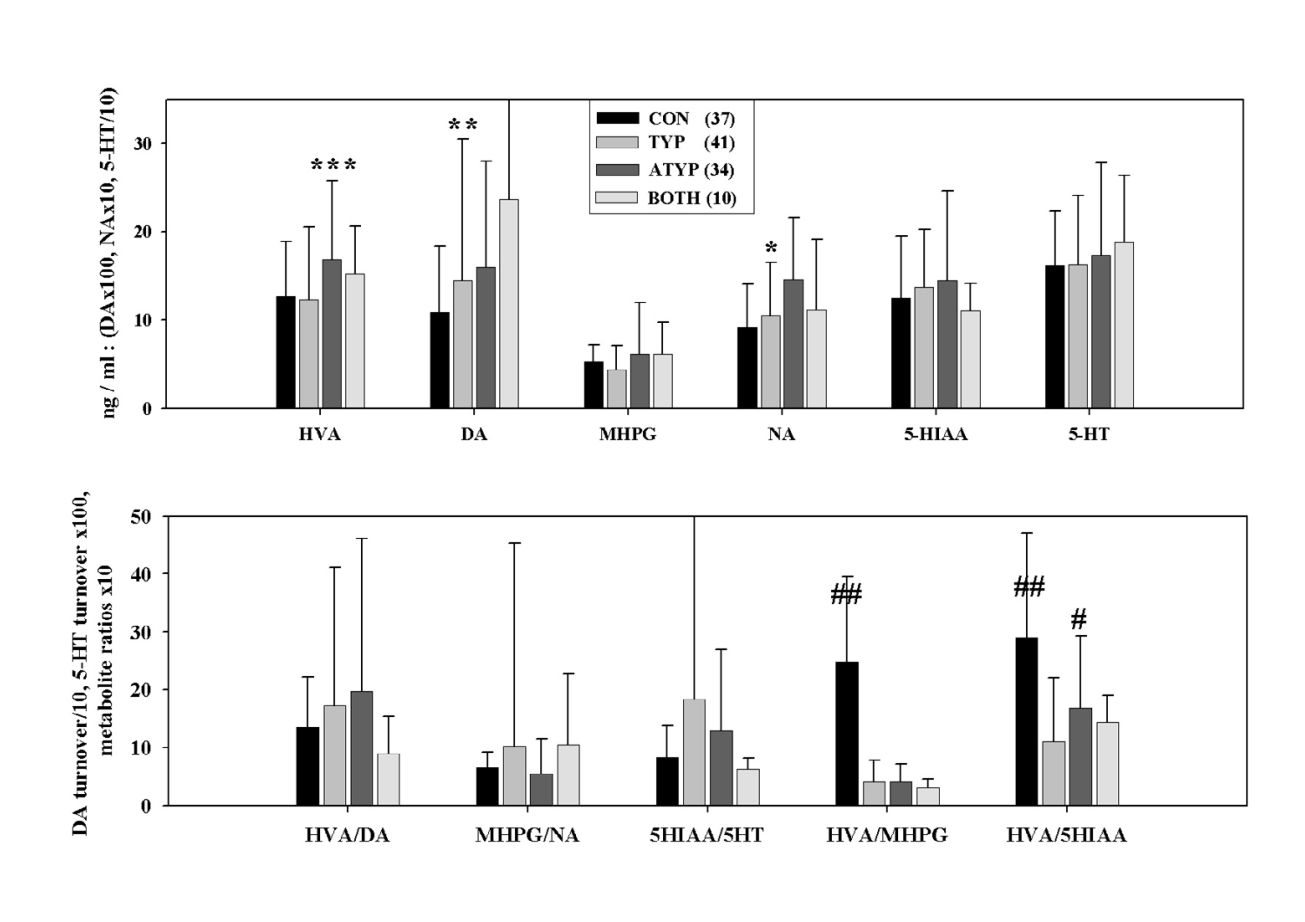
(Top) Serum monoamine and metabolite levels in controls (CON) and in patients treated with typical (TYP), atypical (ATYP) or both types of antipsychotic drug. Atypical > Typical, * *P *< 0.1; ** *P *< 0.08; *** *P *< 0.04; # *P *< 0.006 (covaried for age, IQ and CPZ). (Bottom) Turnover rates for 3 monoamines, and the ratio of dopamine (DA) to noradrenaline (NA) and serotonin (5-HT) metabolism (HVA/MHPG, HVA/5-HIAA), respectively. Controls (*vs. *whole patient group) showed less 5-HT TR (*P *< 0.05) and more HVA *vs. *5-HIAA and MHPG (both ## *P *< 0.002).

**Figure 2:**
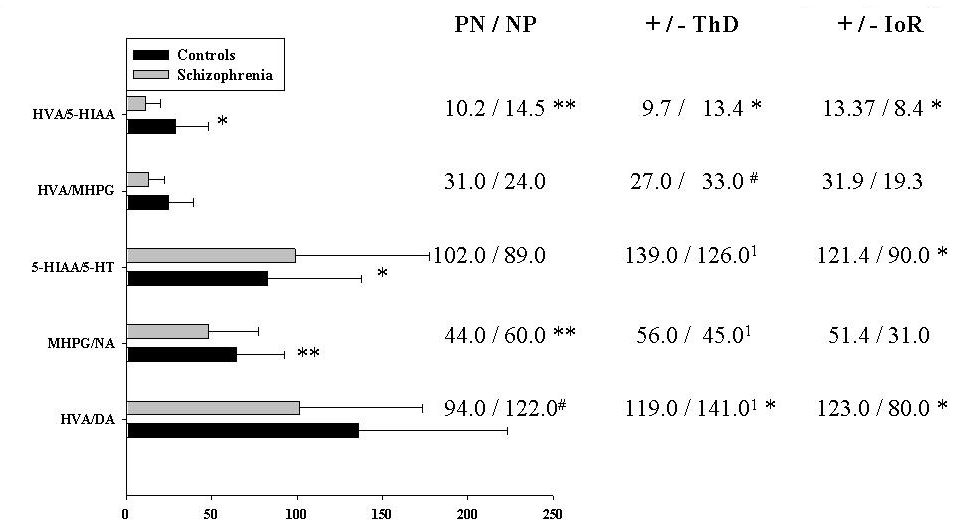
Metabolite/monoamine turnover ratios for the whole subject group (bar diagram, left) and for patient sub-groups (tabular, right: PN, paranoid; NP, nonparanoid; ThD, thought disorder [+/-, high/low]; IoR, ideas of reference [+/-, high/low]). #*P *< 0.08, *P < 0.05, ** *P *< 0.01. 1 = levels of metabolite. Units: inter-amine ratios ×10; 5-HT TR ×10^3^; DA and NA TR ×10.

For the patient group as a whole, turnover for 5-HT was higher, for NA lower, and for DA there was no difference compared to controls. The HVA/5-HIAA ratio was lower in patients but along with catecholamine metabolites, levels increased and normalised more on atypical than typical antipsychotic drug treatment (Fig. 1). There was a trend for the HVA/MHPG ratio to be lower in the patient group (Fig. 2 left).

Thought-disordered (disorganised) patients were characterised by low levels of HVA, HVA/5-HIAA and HVA/MHPG ratios, whereas high DA and 5-HT turnover and a high HVA/5-HIAA ratio were features of those with ideas-of-reference (IoR: Fig. 2 right). Nonparanoid patients showed a much higher NA turnover than the paranoid group. This was reflected in a trend for an increased DA turnover, where the HVA may have partly derived from NA metabolism. The increased NA turnover parallels plasma MHPG levels in patients with negative symptoms and the deficit-syndrome [55]. The non-paranoid group also had a larger HVA/5-HIAA ratio (Fig. 2 centre). We reported previously that increased DA D2 occupancy was predicted by decreases of DA metabolism and of HVA/5-HIAA ratios, especially in paranoid patients. DA D2 occupancy and catecholamine metabolism were unrelated in nonparanoid patients [32].

### Neuropsychology: Noradrenergic Activity

Patients were impaired in the performance of all tests except the Mooney-faces-closure test [[33]: table 1]. Low NA turnover, attributable to low levels of the metabolite MHPG, was characteristic of the patient group as a whole (Fig. 1), but was a significant feature for the paranoid *vs*. the nonparanoid subgroup ([11] Fig. 2 centre).

**Figure 3:**
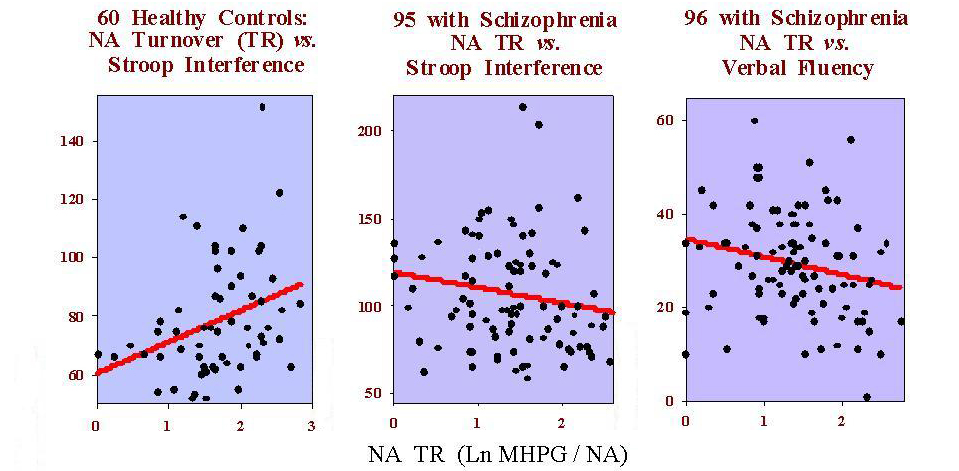
Partial correlations (left) for increased noradrenaline turnover (NA TR: Ln MHPG/NA) with increased Stroop-test interference scores in healthy controls (r = +0.34, *P *= 0.009), and (middle) for decreased NA TR with increased Stroop interference scores in patients with schizophrenia (r = -0.26, *P *= 0.01). But, (right) decreases of NA TR are associated with better verbal fluency in patients with schizophrenia (r = -0.29, *P *= 0.004).

A regression analysis showed significant effects of NA turnover on Stroop-interference, and verbal fluency, with opposite effects evident for controls and patients. For controls (n, 60) increases of Stroop-interference were associated with increases of NA turnover (*F*_1,58 _= 7.4, *P *= 0.009: *r *= +0.34, R^2 ^= 11.3%: Fig. 3, left). The inverse effect, (increasing interference was associated with decreasing NA turnover), was evident for the patients (n, 95: partial correlation *r *= -.26, *P *< 0.01, Fig. 3 middle). However, increased verbal fluency also remained in the last step of this regression, and was clearly associated with decreases of NA turnover (F_2,92 _= 5.5, *P *= 0.005; *r *= -0.29, *P *= 0.004, Fig. 3 right). Together this explained 10.7% of the variance (R^2^). In other words, stimulation of the low levels of NA TR in patients would be expected to improve (reduce) interference in the incongruent Stroop condition but to impair (reduce) verbal fluency.

There were no significant regression models predicting relationships for NA turnover with neuropsychological performance in paranoid patients (n 67) or the sub-groups with marked IoR (n 39) and with little thought disorder (n 43).

However, the non-paranoid (n 28), thought-disordered subgroups (n 51), and patients with few IoR (n 55) showed very similar negative associations for NA activity with verbal fluency. Thus, it was the nonparanoid, thought disordered patients who were responsible for the same effect seen with the patient group as a whole (Table 2 left). Nonparanoid patients with few IoR also showed improving immediate verbal recall as delayed verbal recall deteriorated with decreasing NA activity (Table 2 middle).

**Table 2:** Partial correlations for performance on 4 neuropsychological tasks reflecting left hemisphere (and cingulate) and two reflecting right hemisphere function with NA turnover in 3 patient sub-groups with few paranoid symptoms

		Left hemisphere function								Right hemisphere function				
		Verbal		Stroop		Immediate		Delayed		Trails B – A		Delayed		
		Fluency		Interference		Verbal Recall				Attention Set		Visual Reproduction		
	p	r	p	r	p	r	p	r	p	r	p	r	p	R^2^
**NP **n 28														
F(3,24) = 8.5,	0.0005	-0.60	0.001	--	--	-0.50	0.01	+0.60	0.001	--	--	--	--	51.4%
**High-ThD **n 51														
F(3,47) = 5.1,	0.004	-0.44	0.002	-0.34	0.017	--	--	--	--	--	--	+0.29	0.04	24.6%
**Low-IoR **n 55														
F(5,49) = 4.7,	0.0014	-0.39	0.015	-0.33	0.005	-0.35	0.013	+0.42	0.002	+0.34	0.015	--	--	32.3%

PN = paranoid, NP = Nonparanoid, High-ThD = above median summed thought disorder ratings, Low-IoR = below median of summed ideas-of-reference ratings

Like the patient group as a whole, thought-disordered patients with few IoR showed increasing Stroop interference with decreasing NA turnover. Their right hemisphere functions such as set shifting latency (trails) and nonverbal recall were positively related to NA activity (Table 2). As patients were showing lower than normal NA activity, this means that decreases of NA turnover were associated with poorer delayed recall. Short-term information processing appeared to be enhanced by low NA activity (e.g. verbal productivity and immediate verbal recall in nonparanoid and thought disordered patients), but Stroop and Trails indicators of set-shifting were associated in opposite ways by changes of NA activity in patients with few IoR.

### Neuropsychology: Dopaminergic Activity

The patients as a whole did not differ significantly from the healthy subjects on measures of DA turnover (Fig. 1: [11]). But a trend for less DA activity in patients became significant for the paranoid group (*vs. *nonparanoid). In contrast, patients with marked IoR showed a higher DA turnover than the other subgroups (Fig. 2).

**Figure 4:**
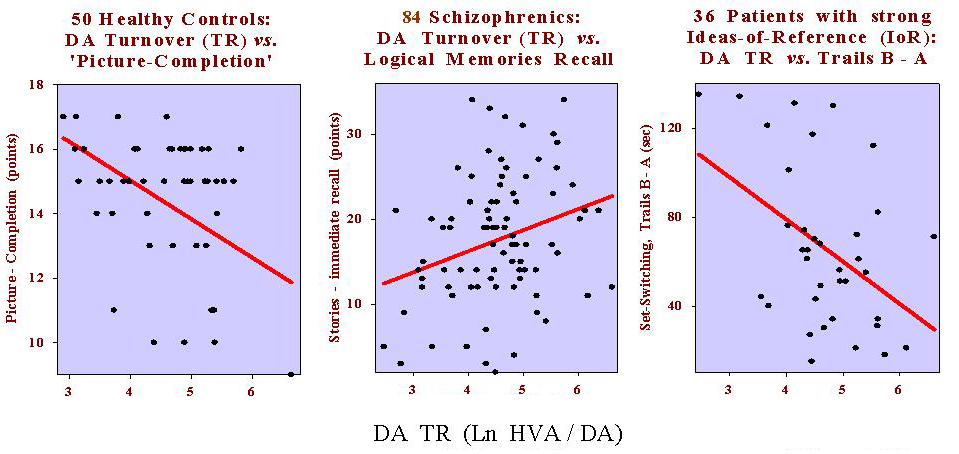
Partial correlations (left) show that reduced dopamine turnover (DA TR: Ln HVA/DA) is associated with enhanced picture completion scores in healthy subjects (r = -0.35, *P *= 0.01). Increasing DA TR (middle) is associated with improved verbal recall in patients with schizophrenia (r = +0.29, *P *= 0.008). For patients with high ratings for ideas-of reference (IoR: right) increased DA activity was associated with promoting the speed of switching between sets on the trail-making test (r = +0.34, *P *= 0.015).

For healthy controls (n 50) only the performance on the picture completion task predicted DA turnover. Better performance was associated with less DA activity (*F*_1,48 _= 6.8, *P *= 0.012, *r *= -0.35, R^2 ^= 12.4%: Fig. 4 left). For the patient group as a whole (n 84) the dominant relationship for DA turnover was with the *immediate *recall of stories. The effect was the opposite of that described for NA turnover where *immediate *recall improved with decreasing NA activity (above). Here, poor *immediate *recall was associated with low, decreasing DA activity (*F*_1,82 _= 7.4, *P *= 0.008, *r *= +0.29, R^2 ^= 8.2%: Fig. 4 middle).

A further relationship of low DA turnover with poor verbal recall was characteristic of those patients expressing little thought-disorder and few IoR (Table 2). The one striking relationship for increased DA turnover was in the IoR subgroup. They tended to show high levels of DA activity. Here increased DA activity was associated with increased set-switching as indicated by decreased Trails B-A differences (Fig. 4, right).

### Neuropsychology: Ratio of Catecholamine Metabolites (Activity)

In controls, relative catecholamine activity (HVA/MHPG) was unrelated to task performance. For the patients as a whole (n 85) the opposing nature of the associations of cognitive measures with DA and NA activity noted above was reflected in a trend (*F*_7,77 _= 1.8, *P *= 0.09, R^2 ^= 14.1%). This showed opposite influences on the right hemisphere functions of immediate and delayed visual reproduction (*r *= +0.23, *P *= 0.04, *r *= -0.25, *P *= .03) and block-design (*r *= + 0.22, *P *= 0.05).

Interestingly these right hemisphere functions reflected the contributions of activity in the high IoR but nonparanoid subgroups (Table 3, right). In addition, in the high IoR group negative relationships emerged for the left hemisphere functions of verbal fluency and cingulate mediation of Stroop interference (Table 3, left). As the DA activity was relatively high in the high-IoR group, increases of the ratio HVA/MHPG may be considered as related to diminishing fluency, yet improved reduction of interference.

**Table 3:** Partial correlations for performance on 4 neuropsychological tasks reflecting left hemisphere (and cingulate) function and 4 tasks reflecting right hemisphere function with DA turnover (**A**: DA TR) and the ratio of DA to NA activity (**B**: HVA/MHPG) in patient subgroups with few paranoid symptoms

		Left hemisphere function								Right hemisphere function								
		Verbal		Stroop		Immediate		Delayed		Block		Trails B-A		Mooney		Delayed		
		Fluency		Interference		Verbal Recall				Design		Attention Set		Faces		Visual Reproduction		
	p	r	p	r	p	r	p	r	p	r	p	r	p	r	p	r	p	R^2^
**a/ DA TR**																		
**NP **n 28																		
F(1,26) = 5.6,	0.026	--	--	--	--	--	--	--	--	--	--	--	--	-0.42	0.026	--	--	16.3%
**Low-ThD **n 41																		
F(1,39) = 8.7,	0.005	--	--	--	--	+0.43	0.005	--	--	--	--	--	--	--	--	--	--	18.3%
High-ThD n 43	N.S.	--	--	--	--	--	--	--	--	--	--	--	--	--	--	--	--	--
**Low-IoR **n 48																		
F(1,46) = 3.9,	0.05	--	--	--	--	+0.28	0.05	--	--	--	--	--	--	--	--	--	--	7.9%
High-IoR n 36																		
F(1,34) = 8.3,	0.007	--	--	--	--	--	--	--	--	--	--	-0.44	0.007	--	--	--	--	19.6% -

**b/HVA/MHPG**																		
**NP **n 25																		
F(1,23) = 5.0,	0.036	--	--	--	--	--	--	--	--	+0.42	0.036	--	--	--	--	--	--	17.8%
**Low-ThD **n 45																		
F(1,48) = 4.7,	0.037	--	--	--	--	--	--	+0.33	0.037	--	--	--	--	--	--	--	--	10.9%
High-ThDn 45	N.S.	--	--	--	--	--	--	--	--	--	--	--	--	--	--	--	--	--
**Low-IoR **n 47																		
F(3,43) = 2.3,	0.095	+0.30	0.047	--	--	--	--	--	--	--	--	--	--	--	--	--	--	13.6%
High-IoR n 38																		
F(4,33) = 4.7,	0.004	-0.35	-0.35	-0.50	0.002	--	--	--	--	+0.50	0.002	--	--	--	--	-0.36	0.03	36.3%

PN = paranoid, NP = Nonparanoid, Low-/High-ThD/IoR = below /above a median split of thought disorder/ideas-of reference ratings. NS = Model not significant

### Neuropsychology: Serotonergic Activity

The patient group showed a slight but significantly higher level of 5-HT activity than the control group ([11] Fig. 2). But among the subgroups only those expressing many IoR showed more 5-HT activity than patients with few IoR. Neither the patient nor the control group, neither the paranoid nor the nonparanoid subgroup showed a model where a significant part of the 5-HT turnover was explained by neuropsychological task performance.

The only patients for whom 5-HT played a significant role in task performance were those who expressed little thought-disorder. This is of interest, firstly as 5-HT metabolism has been reported to play a role in the task performance of disorganised patients elsewhere (see discussion), and secondly because the task was also influenced by catecholamine metabolism. For patients without much thought disorder immediate verbal recall related positively to 5-HT activity (Table 4). There was no significant regression model for patients showing much thought disorder nor for those with a rather high 5-HT turnover (the high IoR subgroup).

**Table 4:** Partial correlations for performance on 4 neuropsychological tasks reflecting left hemisphere (and cingulate) function and 2 tasks reflecting right hemisphere function with 5-HT turnover (**A**: 5-HT TR) and the ratio of DA to 5-HT activity (**B**: HVA/5-HIAA) in patient sub-groups

		Left hemisphere function				Right hemisphere function											
		Verbal		Immediate		Picture		Mooney		Trails B – A		Immedate		Delayed		
		Fluency		Verbal Recall		Completion		Faces		Attention Set		Visual Reproduction				
	p	r	p	r	p	r	p	r	p	r	p	r	p	r	p	R^2^
**a/ 5-HT TR**																
**NP **n 26	N.S.	--	--	--	--	--	--	--	--	--	--	--	--	--	--	--
PN n 70	N.S.	--	--	--	--	--	--	--	--	--	--	--	--	--	--	--
**Low-ThD **n 46																
F(1,44) = 4.3	0.04	--	--	+0.30	0.04	--	--	--	--	--	--	--	--	--	--	8.8%
High-ThD n 49	N.S.	--	--	--	--	--	--	--	--	--	--	--	--	--	--	--
**Low-IoR **n 55	N.S.	--	--	--	--	--	--	--	--	--	--	--	--	--	--	--
High-IoR n 40	N.S.	--	--	--	--	--	--	--	--	--	--	--	--	--	--	--

**b/ HVA/5-HIAA**																
**NP **n 26	N.S.	--	--	--	--	--	--	--	--	--	--	--	--	--	--	--
PN n 59																
F(1,57) = 4.1,	0.04	--	--	--	--	--	--	+0.26	0.04	--	--	--	--	--	--	6.7%
**Low-ThD **n 41																
F(5,35) = 3.5,	0.01	+0.36	0.028	--	--	-0.40	0.01	--	--	--	--	+0.45	0.005	-0.29	0.07	33.3%
High-ThD n 43																
F(1,41) = 4.7,	0.035	--	--	--	--	--	--	+0.32	0.035	--	--	--	--	--	--	10.4%
**Low-IoR **n 53	N.S.	--	--	--	--	--	--	--	--	--	--	--	--	--	--	--
High-IoR n 31																
F'F(4,26) = 5.7,	0.002	--	--	--	--	--	--	+0.51	0.005	-0.50	0.006	+0.53	0.004	-0.48	0.009	46.7%

PN = paranoid, NP = Nonparanoid, Low-/High-ThD/IoR = below /above a median split of thought disorder/ideas-of reference ratings. NS = Model not significant

### Neuropsychology: Ratio of DA to 5-HT Metabolites (Activity)

Was the relative activity of DA to 5-HT (HVA/5-HIAA) associated with any type of cognitive performance? In the controls (n 51), with a higher ratio than in the patients ([11] Fig. 1), HVA/5-HIAA was associated negatively with delayed visual reproduction (*F*_1,49 _= 10.2, *P *= 0.002, *r *= -0.41, R^2 ^= 17.2%). Picture-completion, a measure of right hemisphere function, was also negatively related to DA activity, but there was no direct influence of 5-HT activity. The implication is that normal right hemisphere recall mechanisms are driven by DA activity and mildly modulated by 5-HT activity.

In the whole patient group, where low HVA/5-HIAA ratios tended to normalise on atypical (*vs. *typical) antipsychotic medication (Fig. 1), the relative activity of DA to 5-HT systems was related clearly to the performance on no less than 4 tasks (F_5,79 _= 3.5, *P *= 0.006). The one is characteristic of left hemisphere function (verbal fluency: *r *= +0.23, *P *= 0.04), while the other 3 tasks reflect right hemisphere function (Mooney-faces; r +0.26, *p *= 0.02: immediate visual reproduction; *r *= +0.31, *P *= 0.005: delayed visual reproduction; *r *-0.24, *P *= 0.03).

Analyses of the sub-groups showed that marked positive associations with the Mooney-faces task were contributed by patients who were paranoid, with many IoR (Table 4, bottom). Other right hemisphere functions where HVA/5-HIAA was important (e.g. visual reproduction and picture completion) were found in patients with many IoR but little thought disorder (Table 4). Striking is the large degree of variance explained for non-verbal memory processes, Mooney faces recognition and trails' measures of set-shifting in patients with marked IoR (whose HVA/5-HIAA ratios were significantly above those with few IoR).

## Discussion

This is the first comprehensive report of potential functionally-related associations between neuropsychological performance and plasma markers of the overall activity of 3 monoamines in normal subjects and those with schizophrenia. Novel is that the regression analyses take account of the heterogeneity of schizophrenia, whereby 3 major dimensions of the clinical presentation of schizophrenia were examined: paranoid-nonparanoid, disorganisation/thought-disorder and ideas-of-reference. The tasks represented cognitive contributions of the frontal or temporal lobes in the left or right hemispheres. We now draw together profiles with respect to the variance explained from the perspective of nosological, neuropsychological and biochemical descriptors.

### On the variance explained

First we consider the main results from the point of view of the degree of variance explained in the regression analyses. Considering the whole groups of patients and controls, there was no example of neuropsychological performance relating to the variance of 5-HT turnover. This may reflect an over-proportional contribution from peripheral organs [56]. Theoretically, opposite effects in mutually exclusive subgroups could also be responsible. In practice it should be noted that 5-HT metabolism did have a modulatory effect in some subgroup analyses. In contrast, there were examples in both healthy and patient groups of cognitive performance having a predictive relationship for 8–12% of the variance of NA and DA turnover. A correlation with frontal lobe function was evident with NA activity (Stroop and verbal fluency), while DA activity related to posterior temporo-parietal function (picture completion and immediate verbal recall).

With respect to the subgroups of schizophrenia, the greatest amount of variance was explained in analyses showing similar contributions of the performance on 3–4 tasks to the monoaminergic ratio considered. This is not surprising as the widespread innervation by monoaminergic systems would be expected to contribute to functions sensitive to several tasks. The most striking example was with non-paranoid NA turnover as the independent variable. Here 3 left-hemisphere functions contributed over 50% of the variance (verbal fluency, immediate and delayed verbal recall). Three more large contributions to the variance related to the *relative *activity of the monoamines. Two concerned the patients with marked ideas-of-reference (IoR). For HVA/5-HIAA, right-hemisphere function predominated (trails, Mooney-faces, immediate and delayed visual recall: 47% of the variance). This may implicate a more dominant contribution of DA activity in view of the sparse associations for 5-HT turnover (see below). HVA/MHPG also correlated with performance on 4 tasks (36% of the variance). The two right hemisphere tasks may reflect the contribution of DA activity to more posterior parietal and temporal lobe function (block-design and visual delayed recall, see previous paragraph). Tasks reflecting the left-hemisphere (verbal fluency) and midline cingulate function (Stroop) may reflect the dominant role of NA activity, prominent in nonparanoid subjects (see above).

### Profiles – nosological

In paranoid patients the single significant association concerned HVA/5-HIAA and Mooney-faces performance. This positive association contrasts with the negative relationship with DA activity seen in nonparanoid subjects for Mooney faces. The contrast points to the potential negative influence of increasing DA activity (levels were higher in the nonparanoid group), and the positive influence of increasing HVA/5-HIAA ratios with atypical antipsychotic drugs (that benefited the paranoid patients in particular). Further the paranoid/nonparanoid contrast illustrates well a point made in the introduction: namely, the whole patient group did not show impaired performance on the Mooney faces task (Table 1) despite clearly opposing tendencies in the constituent sub-groups. Otherwise nonparanoid patients were characterised by a) the absence of a relationship between cognitive function and 5-HT activity, where a previous report found low activity related to slow discrimination learning [57], and b) a strong relationship with NA activity (nonparanoid> paranoid, Fig. 3), where increases related to improved delayed verbal recall, but to less verbal productivity – that is usually poor in this subgroup [58].

Like the nonparanoid group, patients with little thought-disorder showed a relationship between frontal functions and NA activity. But they also showed a relationship of the performance on 5 tasks to an aspect of 5-HT activity (turnover or the ratio to DA activity). Indeed one might posit a similar role for the relative strength of DA/5-HT activity in immediate verbal and visual recall, picture completion and even verbal fluency. In contrast those with high levels of thought-disorder had a limited set of associations with left frontal (NA) function and right parietal involvement (HVA/5-HIAA). The relatively high levels of 5-HT activity found in these thought-disordered patients tends to be supported by drug-challenge, genetic and plasma measures [59-61].

For subjects with few or with marked IoR there was a remarkable set of associations between cognition and monoamine activity. For those with few IoR left hemisphere (frontal and temporal lobe) cognitive function related to NA metabolism. But in patients with strong IoR, right hemisphere functions (trails, Mooney-faces, immediate and delayed visual recall) were related to HVA/5-HIAA. It is notable that some of the latter functions incur parietal activity that was reported to be high in imaging studies of patients with marked IoR [62].

### Profiles – monoamines

NA activity correlated with neuropsychologic function in patients with much thought disorder and those who were nonparanoid with few IoR. Decreasing NA metabolism was favourably related to improved verbal fluency, and less Stroop interference. Further the decreases of NA metabolism in nonparanoid patients with few IoR related to better immediate verbal recall. These normalising effects of decreasing NA activity in thought disordered patients concur with reports on plasma levels of MHPG [63], and results achieved on trials of NA antagonists [64,65].

Increases of DA activity related positively to improved immediate verbal recall in those without much thought disorder or IoR. On the other hand increasing DA activity was related to reduced Mooney-faces scores in nonparanoid patients and faster switching of set in the trails task. This is consistent with decreases of switching performance registered after treatment with DA antagonists (e.g. antisaccades, trails, Stroop; [66]). The relative degree of DA to NA activity (i.e. HVA/MHPG) was important for left and right hemisphere function (4 tasks) especially in those with marked IoR (Table 2, bottom).

For 5-HT activity, the important issue for cognitive function was its activity relative to that of DA (HVA/5-HIAA). This may reflect the opposing influences of DA and 5-HT on the processes underlying efficient cognitive function (e.g. working memory, [67]). The importance of the ratio was evident in patients with productive symptoms (paranoid), much thought-disorder and IoR. The direction of the association could be negative or positive, but remarkable is that the functions largely belong to the domain of the right hemisphere (Table 4 bottom).

### Profiles – Neuropsychology

Here we point out only the strongest relationships for the 10 neuropsychological tasks. For verbal fluency and Stroop interference (signs of frontal and cingulate functions) correlations were seen in highly symptomatic patients. Verbal fluency was reduced with increasing NA activity in the thought-disordered and those with negative symptoms (nonparanoid). This seems to parallel the sharp rise (then fall) of negative symptoms and plasma NA levels in a study of the effects of ketamine-induced psychosis [68]. However, they reported associations with impaired verbal memory rather than fluency – although it is difficult to be sure what the state of the NA response was in each task. NA activity was also implicated in those with many IoR where decreasing HVA/MHPG ratios were associated with increased Stroop interference. For those with high IoR, decreasing latencies on the trails B-A task, a frontal sign of the ability to shift between set, related to increasing DA turnover and HVA/5-HIAA ratios. In the same patients worsening block-design and Mooney-faces performance (signs of parietal function) related to decreasing HVA/MHPG and HVA/5-HIAA, respectively. Among the few studies of this patient subgroup, others have noted an association between IoR symptoms and performance on tests of self-monitoring abilities such as these [69], and for ketamine-induced IoR with DA binding activity [70].

A remarkable feature across analyses is that the signs of the correlations for immediate verbal or visual recall usually changed for the delayed recall version (Table 2 left, Table 4 right). Nonetheless poorer immediate verbal recall was associated with decreasing DA turnover in the low thought-disorder group, while poorer delayed verbal recall related to decreasing NA turnover in the nonparanoid patients. One could perhaps generalise this result: poor recall abilities are associated with low catecholamine activity, especially in patients not showing positive and productive symptoms.

### General: methods – advantages/disadvantages

Many previous reports have deliberated over the interpretation of biochemical data collected remotely from the source; CSF, blood (plasma) and urinary measures provide peripheral data remote from the CNS monoamine pathways producing them. Nonetheless, all the products of monoamine metabolism will spill over and be reflected in peripheral catchments on the way to breakdown and excretion. Major psychological or psychiatric states and drug treatment are the most likely agents for changes of transmitter activity from the norm. Schizophrenia and antipsychotic drugs are here widely documented cases in point, where changes of monoaminergic activity clearly affect peripheral and central functions alike.

We argue that the obvious disadvantages of this study in not being able to measure at source are partly counteracted by two innovations. The first is the more precise description of the clinical state. We suggested in the introduction that clinicians easily distinguish the confused thought processes of disorganised patients from those who hallucinate frequently. Further, the clearly separate substrates responsible are not only reflected in different neuropsychological performance and state but reflect different levels of monoaminergic activity. By separating these factors it should be possible to find relationships between cognition and monoamine activity in the direction of those reported here. We limited ourselves to extreme states on the dimensions of paranoia, disorganisation and ideas-of reference (6 groups). Others would do well to study the relations for negative symptoms: (e.g. are plasma HVA levels higher in deficit *vs. *nondeficit schizophrenia [55] or vice versa due to symptom profile [71] or typical antipsychotic treatment [72], or is the type of onset critical (abrupt/gradual) where the age of onset was unrelated to plasma HVA levels [10]?).

The second innovation was the use of turnover measures of metabolism and inter-amine activity ratios. This was intended to resolve the following issue. Reliance on amine measures may, in the case of (say) high levels, reflect increased release resulting from increased metabolic demand, and increased impulse flow [73]. But they may also reflect production over the need, as often seen when circumstances change or the ongoing state inhibits an adaptive reduction of release. With these innovations we proposed to find changes and relationships that have otherwise been masked by the study of subgroups with opposing tendencies within the population under investigation. This may have been the case with reports of the absence of associations found in relatively chronic adult patients [74] and adolescents with a more recent onset [75]. A third important feature of this study helped add precision to a close description of psychiatric state and the general state of monoaminergic activity. This has been the ability through recent neuroimaging and brain-damage studies to relate the above changes of biochemical and psychological state to the function of gross divisions of the CNS in performing well-studied neuropsychological tasks [76].

### General: conflict and consistency in CSF monoamine findings

CSF levels of NA were reported to be unrelated to verbal abilities, arithmetic or memory function [74,75,77]. CSF MHPG levels were not associated with performance on tasks testing memory and executive function [29]. Such negative results are unexpected considering that high NA (potentially reflecting a low turnover) and decreasing MHPG levels in the CSF have been associated with increasing psychopathology [77,78]. But, some apparently conflicting findings [77] are consistent with our results in one aspect. The psychopathology they described related to positive symptoms: it was in such paranoid patients that we found a lower NA turnover. Further the absence of a relationship for NA levels with verbal abilities and memory [77] is explicable by our finding that decreasing NA activity related to improved verbal fluency and recall. The subtle difference is that we found this relationship most evident in patients who suffered much thought disorder or few IoR or paranoid symptoms – those with higher NA turnover.

CSF levels of 5-HIAA have been related to performance on tests of attention, working memory and executive function as measured by the digit-symbol, digit-span and picture arrangement tasks [19,30]. Both groups noted a correlation between HVA and 5-HIAA levels, and the relationship of HVA/5-HIAA to both cognitive measures and symptom ratings [19]. So it is not surprising and supportive of our methods that we also found that a clinical improvement following atypical antipsychotic drug-treatment was associated with increased HVA/5-HIAA ratios [11], and that several cognitive functions (associated with the right hemisphere) improved or worsened with increases and decreases of the HVA/5-HIAA ratio, respectively. Novel was the finding that this relationship was marked for patients with many IoR and little thought disorder in the trails-measure of attention-shift and in visual memory. In contrast, those with thought-disorder and paranoia showed similar relationships but restricted to the Mooney-faces-closure test (Table 4).

It was surprising to find relatively few relationships between DA activity and task performance, considering there was a broad range of high to low values among the healthy subjects and suppressed levels among patients – to which both the illness and medication would have contributed. However, it may be noted that even with other patient groups (e.g. HIV: [79]), where CSF HVA levels were <50% of the non-affected controls, correlations were restricted to response slowing on tests of executive attention and concentration and unrelated to memory performance. For elderly subjects an inverse relationship between plasma HVA and bradykinesia was reported [80], which to a degree is consistent with studies of schizophrenia [29] and schizotypal disorder [17] where more neuropsychological impairment was observed with decreasing levels of CSF HVA. Our finding of a positive relationship with HVA for immediate verbal recall in patients without thought disorder and IoR (Table 2) fits into this pattern, but contrasts with plasma HVA findings in a sample of chronically ill patients [81]. But we agree with a report from the same research group [19], that where there was a lack of association with HVA alone, the ratio HVA/5-HIAA explained performance better. (Note: typical antipsychotic drugs impair performance on many tests of memory [82], but some of the above results were obtained with non-medicated subjects [17] and, in this report with subjects on atypical antipsychotic medication.)

The one intriguing exception to these reports on the effects of decreases of HVA concerned patients showing many IoR. These patients not only showed unusually high levels of DA metabolism but also a correlation of shorter set-shift latencies on the trails' test with increasing DA turnover. This fits well with an early formulation of the role of DA in switching between information processing channels [83] and could form the basis of a working hypothesis for future study of the bases of these patients' illusions. Namely, that the increased DA activity permits switching between multiple associations for a given sensory input, permitting in the pathological case a grossly inappropriate interpretation of incoming information.

In conclusion, the profiles of the associations of neuropsychological performance with changes of activity of dopamine, noradrenaline and serotonin (and their activities relative to each other), can assist with an understanding of the modulation by monoaminergic pathways of frontal and temporal lobe function. This pertains even though the origin of the peripheral monoamine measures cannot be attributed to any particular anatomical source. Since this report concentrates on patients showing strong or few symptoms on three major dimensions of psychopathology, the profiles for these sub-groups of schizophrenia should help to inform goal-directed interventions in the treatment of patients with these particular features.

## Methods

### Subjects

Using DSM-IV criteria [34] 108 patients from the University Psychiatry Clinics were diagnosed with schizophrenia by the senior ward physician and subsequently by 2 senior physicians associated with this research. Affective, schizoaffective and schizophreniform psychoses were excluded. The undifferentiated subtype was regarded as a residual category that contrasts with the paranoid, disorganised and catatonic subtypes. Patients were screened to exclude other major psychiatric or somatic illness, alcohol abuse in the last 5 years and substance abuse other than nicotine at the time of testing. A group of 63 healthy controls, recruited by advertisement and paid for their participation, was closely matched for age, education, socio-economic family status and handedness. Exclusion criteria for controls were the same as for patients, and based on a semi-structured interview: they reported no family history of psychosis, nor that they had ever consulted with a psychiatrist or psychologist. Following approval from the Hospital Ethics Committee, in accord with the Declaration of Helsinki informed signed consent was obtained from each patient and the responsible care-giver, and from each healthy participant. Clinical and demographic data are given in table 1.

The study took place in the post-acute phase, on average 6 weeks after the patient's admission (range 4–8 weeks) and following stabilisation of treatment. Of 101 patients providing biochemical data 99 were treated with antipsychotic drugs according to their clinical requirements. Drug doses were converted to chlorpromazine equivalents (CPZ: [[35-38], correspondence with the suppliers of olanzapine and sertindole]). Patients receiving conventional, atypical or both categories of antipsychotic drug were considered separately and together [[11]: table 1]. Symptoms were rated with the Positive and Negative Syndrome Scale (PANSS: [39], along with Schneiderian ideas-of-reference (ego-disturbance) and thought-disorder items from the Scale for Assessment of Positive Symptoms (SAPS: [6]) that are under-represented in the PANSS. The subtypes of schizophrenia was based on a factor analysis of the symptom ratings. This resulted in 4 dimensions (varimax rotation, eigen values >1, excluding those contributing <5% to the variance: [3]): (1) disorganised (especially thought/concept disorder), (2) nonparanoid (largely negative symptoms), (3) ideas-of-reference and (4) paranoid (positive symptoms). Four categorical groupings were made on the basis of this factor analysis. Thought disorder (ThD) and ideas-of-reference (IoR) ratings were split at the median to provide groups with high *vs*. low levels of the respective symptom clusters. Paralleling the paranoid/nonparanoid (PN/NP) diagnostic split, two mutually exclusive groups with PN and NP symptoms were formed. This procedure resulted in a comparison of 6 subgroups of patients with schizophrenia.

### Neuropsychology

Ten tasks were administered. The verbal-fluency test [40] required the generation of as many words as possible starting with the letter F, A or S (1 minute each). In the trail-making test subjects were asked to join up in sequence first a series of numbers (form-A), then an alternating series of letters and numbers (form-B, e.g. 1-A-2-B-3; the score used was the form-B-minus-A latency; [41]). These tests reflect functions in the left and right frontal lobes, respectively [33]. The Stroop test interference score was the increased latency to name the print colour of a word that named a different colour compared to the latencies to name colours and words naming colours. Performance activates the frontal and cingulate cortices [42,43].

The block-design and Mooney faces closure tests reflect broadly parietal function. Block-design required that a given square form was reconstructed out of 4 or 9 pieces [44]. The modified Mooney faces closure test asked for the classification of the age of degraded images of faces [45,46]. In the picture-completion test (a reflection of temporo-parietal function) the subject identified the missing feature on a picture of an everyday scene [44]. Visual reproduction and logical memories under conditions of immediate- and delayed-recall [47] involved a presentation of a series of visual patterns or two short stories for recall, and reflect right- and left-sided temporal lobe function in visuo-spatial and verbal memory, respectively. In addition the short 12-item form of the Advanced Progressive Matrices (APM) was used as a measure of IQ, where scores <6 are below and scores of 12 are above average [[48]: table 1].

### Serum assessments and assays for monoamines and their metabolites

A 30 ml blood sample was taken at 08.00 (± 30 min) after 10 h of fast and rest before smoking, medication, exercise and breakfast. The sample was centrifuged for 10 min at 2000 g and stored at -70°C until analysis. All samples were analysed blind to their origin by reversed phase high performance liquid chromatography with a glassy carbon electrochemical detector using internal standards. Separate isocratic determinations were run for a) DA and NA, b) 5-HT with organic-sodium dihydrophosphate mobile phases [after [49]], c) HVA, 5-HIAA, [50] and d) MHPG with organic sodium-acetate mobile phases [51]. Intra- and inter-assay coefficients of variation were, DA 11.6/8.9%, NA 11.6/9.1%, 5-HT 2.3/9.2%, HVA 7.1/19.8%, 5-HIAA 6.0/7.3% and MHPG 6.9/18.1%, respectively. Recovery ranged from ca. 74–80% with the following sensitivities (ng/mL) for DA 0.5, NA 0.01, 5-HT 1.0, HVA and 5-HIAA 1.25, and MHPG 1.0.

Serotonergic measures allowed for the taking of comparable metabolic measures between monoamines. Circulating levels can be expressed in terms of platelet numbers, their protein content, or per unit volume of blood, serum or platelet-rich plasma. The first two methods may be contaminated by non-platelet derivatives and their proteins, while the latter 3 measures can vary with efflux from the platelets. Nonetheless, a high correlation between platelet and circulating levels with excellent intra-individual replicability at 3 months has been repeatedly reported for healthy subjects [52,53]. Our values match those of Jernej [53] and 5 studies discussed (98–312 ng/ml), and are a bit lower than in 3 reports on platelet-rich plasma and serum (269–271 ng/ml). Turnover rates were similar to plasma values [54]. The scarcity of outliers (± 2 SD) was similar between the monoamines, does not point to irregular platelet-release and emphasises the lack of variability.

### Data treatment

The biochemical data from 5 subjects were above a cut-off criterion for outliers of the mean ± 2 SD and removed from the analysis. For technical reasons some data were missing from individual measures and covariates for 15 subjects. This resulted in full sets of data across all measures in 73 patients and 37, less a further 10 patients for the metabolite ratios. Their demographic and clinical characteristics did not differ from the original sample [32]. The number of subjects used in each analysis is cited below in parentheses. Normal distributions of the biochemical data were obtained after natural logarithmic transformations.

Separate multivariate analyses of covariance were used to compare task performance or biochemical measure between subject groups. Age and IQ were related to some biochemical measures, and therefore used as covariates. Other demographic variables (e.g. education, status) were not used: they were matched between groups, their variance was not large and their communal contribution overlapped with age and IQ. Biochemical data were analysed in two stages. The first considered the 3 monoamines and their 3 metabolites [11]. The second, forming the basis for the present report, concerned the 3 turnover ratios and two inter-amine metabolite ratios (e.g. HVA/5-HIAA, HVA/MHPG). The differential effect of conventional and atypical medication was controlled for the effect of dose with the use of chlorpromazine equivalents (CPZ) as covariate. The locus of effect was sought by t-tests (patient data) and one-way analyses of variance (neuropsychology and biochemistry). The relationships of clinical subgroups to biochemical measures, and neuropsychological performance were reported in Oades et al. [11,32,33] and are described here only in summary form.

The emphasis in the present report lies with an explanation of the contribution to test performance by the groups and subgroups of patients and the controls made by measures of plasma monoamine activity with the use of standard, linear regression models. First task performances (n 10) were entered into equations for turnover measures in the patient and control groups. Following significant standard regression models at α = 0.01, the relationships for the 6 sub-groups of patients for explanation by 5 measures of biochemical ratios were explored with backward, step-wise, linear regression models. We emphasise an α-correction to 0.001% for significance. However, in view of the exploratory nature of the study and the inherent uncertainties associated with the models, we describe trends up to levels of <0.05%.

## Competing interests

The author(s) declare that they have no competing interests

## Abbreviations

DA, dopamine, CSF cerebrospinal fluid, HVA homovanillic acid, IoR ideas-of-reference, MHPG 3-methyl-4-hydroxyphenylglycol, NA noradrenaline, NP nonparanoid, PN paranoid, ThD thought-disorder, 5-HT serotonin, 5-HIAA 5-hydroxyindoleacetic acid,

## References

[B1] Bleuler E (1911). Dementia Praecox oder Gruppe der Schizophrenien. Vienna, Deuticke.

[B2] Carpenter WT, Kirkpatrick B, Buchanan RW (1999). Schizophrenia: syndromes and diseases. J Psychiatr Res.

[B3] Bender S, Müller B, Sartory G, Oades RD (2001). Conditioned blocking and schizophrenia: a replication and study of the role of symptoms, age, onset-age of psychosis and illness-duration. Schizophr Res.

[B4] Amin F, Davidson M, Kahn RS, Schmeidler J, Stern R, Knott PJ, Apter SH (1995). Assessment of the central dopaminergic index of plasma HVA in schizophrenia. Schizophr Bull.

[B5] Amin F, Silverman JM, Siever LJ, Smith CJ, Knott PJ, Davis KL (1999). Genetic antecedents of dopamine dysfunction in schizophrenia. Biol Psychiatry.

[B6] Andreasen NC, Olsen S (1982). Negative versus positive schizophrenia: definition and validation. Arch Gen Psychiatry.

[B7] Galinowski A, Poirier MF, Aymard N, Leyris A, Beauverie P, Bourdel MC, Loo H (1998). Evolution of plasma homovanillic acid (HVA) in chronic schizophrenic patients treated with haloperidol. Acta Psychiatr Scand.

[B8] Garver DL, Steinberg JL, McDermott BE, Yao JK, Ramberg JE, Lewis S, Kingsbury SJ (1997). Etiologic heterogeneity of the psychoses: is there a dopamine psychosis?. Neuropsychopharmacology.

[B9] Zhang ZJ, Peet M, Ramchand CN, Shah S, Reynolds GP (2001). Plasma homovanillic acid in untreated schizophrenia – relationship with symptomatology and sex. J Psychiat Res.

[B10] Sharma RP, Javaid JI, Davis JM, Janicak PG (1998). Pretreatment plasma homovanillic acid in schizophrenia and schizoaffective disorder: the influence of demographic variables and the inpatient drug-free period. Biol Psychiatry.

[B11] Oades RD, Klimke A, Henning U, Rao ML (2002). Relations of clinical features, subgroups and medication to serum monoamines and schizophrenia. Hum Psychopharmacol.

[B12] Pliszka SR, Maas JW, Javors MA, Rogeness GA, Baker J (1994). Urinary catecholamines in attention-deficit hyperactivity disorder with and without comorbid anxiety. J Am Acad Child Adolesc Psychiatry.

[B13] Bowers MB, Swigar ME, Jatlow PI, Goicoechea N (1984). Plasma catecholamine metabolites and early response to haloperidol. Clin Psychiatry.

[B14] Bowers MB, Swigar ME, Jatlow PI, Hoffman FJ, Goicoecha N (1987). Early neuroleptic response: clinical profiles and plasma catecholamine metabolites. J Clin Psychopharmacol.

[B15] Sharma RP, Javaid JI, Janicak PG, Faull K, Comaty J, Davis JM (1989). Plasma and CSF HVA before and after pharmacological treatment. Psychiat Res.

[B16] Siever LJ, Tamminga CA, Schulz SC (1991). The biology of the boundaries of schizophrenia. Schizophrenia: Advances in Neuropsychiatry and Psychopharmacology.

[B17] Siever LJ (1994). Biologic factors in schizotypal personality disorders. Acta Psychiat Scand.

[B18] Scheepers FE, Gispen-De-Wied CC, Westenberg HG, Kahn RS (2001). The effect of olanzapine treatment on monoamine metabolite concentrations in the cerebrospinal fluid of schizophrenic patients. Neuropsychopharmacology.

[B19] Forrest TJ, Allen DN, Weiner C, Yao JK, Gurklis JA, Van Kammen DP (2001). The HVA/5-HIAA ratio and cognitive function in schizophrenia. Schizophr Res.

[B20] Wieselgren I-M, Lindström LH (1998). CSF levels of HVA and 5-HIAA in drug-free schizophrenic patients and healthy controls: a prospective study focused on their predictive value for outcome in schizophrenia. Psychiat Res.

[B21] Kahn RS, Davidson M, Knott P, Stern RG, Apter SH, Davis KL (1993). Effect of neuroleptic medication on cerebrospinal fluid monoamine metabolite concentrations in schizophrenia: serotonin-dopamine interactions as a target for treatment. Arch Gen Psychiatry.

[B22] Maas JW, Bowden CL, Miller AL, Javors MA, Funderburg LG, Berman N, Weintraub ST (1997). Schizophrenia, psychosis and cerebral spinal fluid homovanillic acid concentrations. Schizophr Bull.

[B23] Butler PD, Harkavy-Friedman JM, Amador XF, Gorman JM (1996). Backward masking in schizophrenia: relationship to medication status, neuropsychological functioning, and dopamine metabolism. Biol Psychiatry.

[B24] Waldo MC, Adler LE, Leonard S, Olincy A, Ross RG, Harris JG, Freedman R (2000). Familial transmission of risk factors in the first-degree relatives of schizophrenic people. Biol Psychiatry.

[B25] Bender S, Schall U, Wolstein J, Grzella I, Zerbin D, Oades RD (1999). A topographic event-related potential follow-up study on 'prepulse inhibition' in first and second episode patients with schizophrenia. Psychiat Res Neuroimaging.

[B26] Oades RD, Bender S, Schall U, Klimke A, Balcar A, Thienel R (1999). Improved indices of attention in schizophrenia predicted by increased serotonin metabolism: admission /discharge comparisons in first/second episode patients. Psychophysiology.

[B27] Oades RD, Müller B, Schall U, Bender S, Wolstein J (2000). Automatic vs. controlled attention in schizophrenia: conditioned blocking and sensory gating. Behav Pharmacol.

[B28] Waldo MC, Gerhardt G, Baker NJ, Drebing C, Adler LE, Freedman R (1992). Auditory sensory gating and catecholamine metabolism in schizophrenic and normal subjects. Psychiat Res.

[B29] Kahn RS, Harvey PD, Davison M, Keefe RSE, Apter SH, Neale JM, Mohs RC, Davis KL (1994). Neuropsychological correlates of central monoamine function in chronic schizophrenia: relationship between CSF metabolites and cognitive function. Schizophr Res.

[B30] Csernansky JG, King RJ, Faustman WO, Moses JA, Poscher ME (1990). 5-HIAA in cerebrospinal fluid and deficit schizophrenic characteristics. Br J Psychiatry.

[B31] Zakzanis KK, Kaplan E, Leach L (1999). Neuropsychological differential diagnosis. Lisse, The Netherlands, Swets & Zeitlinger.

[B32] Oades RD, Rao ML, Bender S, Sartory S, Müller BW (2000). Neuropsychological and conditioned blocking performance in patients with schizophrenia: assessment of the contribution of neuroleptic dose, serum levels and dopamine D_2_-receptor occupancy. Behav Pharmacol.

[B33] Oades RD, Müller BW, Bender S, Sartory G (2001). Neuropsychological indicators of heteromodal cortex (dys)function relevant to the expression and impairment of conditioned blocking measures of selective attention in schizophrenia. Cogn Neuropsychiatry.

[B34] American Psychiatric Association (1994). Diagnostic and statistical manual of mental disorders: DSM-IV.

[B35] Benkert O, Hippius H (1986). Psychiatrische Pharmakotherapie.

[B36] Rey M-J, Schulz P, Costa C, Dick P, Tissot R (1989). Guidelines for the dosage of neuroleptics. 1: Chlorpromazine equivalents of orally administered neuroleptics. Int Clin Psychopharmacol.

[B37] Schulz P, Rey M-J, Dick P, Tissot R (1989). Guidelines for the dosage of neuroleptics. II: Changing from daily oral to long-acting injectable neuroleptics. Int Clin Psychopharmacol.

[B38] Kane JM (1996). Drug therapy: Schizophrenia. New England J Med.

[B39] Kay SR, Opler LA, Fizbein A (1992). The Positive and Negative Syndrome Scale (PANSS).

[B40] Benton AL, Hamsher K (1989). Multilingual Aphasia Examination.

[B41] Reitan RM (1958). The validity of the Trail Making Test as an indicator of organic brain damage. Percept Mot Skills.

[B42] Leung H-C, Skudlarski P, Gatenby JC, Peterson BS, Gore JC (2000). An event-related functional MRI study of the Stroop color word interference task. Cereb Cortex.

[B43] MacDonald AW, Cohen JD, Stenger VA, Carter CS (2000). Dissociating the role of the dorsolateral prefrontal and anterior cingulate cortex in cognitive control. Science.

[B44] Wechsler D (1981). Wechsler Adult Intelligence Scale-revised (WAIS-R).

[B45] Lansdell H (1970). Relation of extent of temporal removals to closure and visuomotor factors. Percept Mot Skills.

[B46] Mooney CM, Ferguson GA (1951). A new closure test. Canad J Psychol.

[B47] Wechsler D (1987). The Wechsler Memory Scale-Revised San Antonio, TX.

[B48] Raven JC, Kratzmeier H, Horn R (1976). Raven-Matrizen-Test: Advanced Progressive Matrices German language manual.

[B49] Eriksson B-M, Persson B-A (1982). Determination of catecholamines in rat heart tissue and plasma samples by liquid chromatography with electrochemical detection. J Chromatogr.

[B50] Gypta RN, Whelton C (1992). Determination of plasma homovanillic acid by liquid chromatography with electrochemical detection. J Chromatogr.

[B51] Minegishi A, Ishizaki T (1984). Determination of free 3-methoxy-4-hydroxy-phenylglycol with several other monoamine metabolites in plasma by high-performance liquid chromatography with amperometric detection. J Chromatogr.

[B52] Croonenberghs J, Delmeire L, Verkerk R, Lin AH, Meskal A, Neels H, Van der Plancken M, Scharpe S, Deboutte D, Pison G, Maes M (2000). Peripheral markers of serotonergic and noradrenergic function in post-pubertal, Caucasian males with autistic disorder. Neuropsychopharmacology.

[B53] Jernej B, Banovic M, Cicin-Sain L, Hranilovic D, Balija M, Oreskovic D, Folnegovic-Smalc V (2001). Physiological characteristics of platelet/circulatory serotonin: study on a large human population. Psychiat Res.

[B54] Dursun SM, Whitaker RP, Andrews H, Reveley MA (1997). Effects of natural ageing on plasma 5-HT turnover in humans. Hum Psychopharmacol.

[B55] Thibaut F, Ribeyre J-M, Dourmap N, Menard J-F, Dollfus S, Petit M (1998). Plasma 3-methoxy-4-hydroxyphenylglycol and homovanillic acid measurements in deficit and non-deficit forms of schizophrenia. Biol Psychiatry.

[B56] Lambert GW, Kaye DM, Cox HS, Vaz M, Turner AG, Jennings GL, Esler MD (1995). Regional 5-hydroxyindoleacetic acid production in humans. Life Sci.

[B57] Oades RD (1997). Stimulus dimension shifts in patients with schizophrenia, with and without paranoid hallucinatory symptoms, or obsessive compulsive disorder: strategies, blocking and monoamine status. Behav Brain Res.

[B58] Himmelhoch S, Taylor SF, Goldman RS, Tandon R (1996). Frontal lobe tasks, antipsychotic medication and schizophrenia syndromes. Biol Psychiatry.

[B59] Soper HV, Elliott RO, Rejzer AA, Marshall BD (1990). Effects of fenfluramine on neuropsychological and communicative functioning in treatment-refractory schizophrenic patients. J Clin Psychopharmacol.

[B60] Malhotra AK, Goldman D, Mazzanti C, Clifton A, Breier A, Pickar D (1998). A functional serotonin transporter (5-HTT) polymorphism is associated with psychosis in neuroleptic-free schizophrenics. Mol Psychiat.

[B61] Duval F, Mokrani MC, Monreal J, Bailey P, Valdebenito M, Crocq MA, Macher JP (2003). Dopamine and serotonin function in untreated schizophrenia: clinical correlates of the apomorphine and d-fenfluramine tests. Psychoneuroendocrinol.

[B62] Frith CD, Blakemore S-J, Wolpert DM (2000). Explaining the symptoms of schizophrenia: abnormalities in the awareness of action. Brain Res Rev.

[B63] Kaneko M, Honda K, Kanno T, Horikoshi R, Manome T, Watanabe A, Kumashiro H (1992). Plasma free 3-methoxy-4-hydroxyphenylglycol in acute schizophrenics before and after treatment. Neuropsychobiol.

[B64] Yorkston NJ, Zaki SA, Weller MP, Gruzelier JH, Hirsch SR (1981). DL-propranolol and chlorpromazine following admission for schizophrenia: a controlled comparison. Acta Psychiat Scand.

[B65] Freedman R, Kirch DG, Bell J, Adler LE, Pecevich M, Pachtman E, Denver P (1982). Clonidine treatment of schizophrenia: double blind comparison to placebo and neuroleptic drugs. Acta Psychiat Scand.

[B66] Nieman DH, Bour LJ, Linszen DH, Goede J, Koelman JHTM, Gersons BPR, Ongeboer de Visser BW (2000). Neuropsychological and clinical correlates of antisaccade task performance in schizophrenia. Neurology.

[B67] Luciana M, Collins PF, Depue RA (1998). Opposing roles for dopamine and serotonin in the modulation of human spatial working memory functions. Cereb Cortex.

[B68] Newcomer JW, Farber NB, Jevtovic-Todorovic V, Selke G, Melson AK, Hershey T, Craft S, Olney JW (1998). Ketamine-induced NMDA receptor hypofunction as a model of a memory impairment and psychosis. Neuropsychopharmacol.

[B69] Stirling JD, Hellewell JSE, Qurashi N (1998). Self-monitoring dysfunction and the schizophrenic symptoms of alien control. Psychol Med.

[B70] Vollenweider FX, Vontobel P, Oye I, Hell D, Leenders KL (2000). Effects of (S)-ketamine on striatal dopamine: a [^11^C]raclopride PET study of a model psychosis in humans. J Psychiatr Res.

[B71] Nibuya M, Kanba S, Sekiya U, Suzuki E, Matsuo Y, Kinishita N, Shintani F, Yagi G, Asai M (1995). Schizophrenic patients with deficit syndrome have higher plasma homovanillic acid concentrations and ventricular enlargement. Biol Psychiatry.

[B72] Suzuki E, Kanba S, Koshikawa H, Nbuya M, Yagi G, Asai M (1996). Negative symptoms in nondeficit syndrome respond to neuroleptic treatment with changes in plasma homovanillic acid concentrations. J Psychiat Neurosci.

[B73] Amin F, Davidson M, Davis KL (1992). Homovanillic acid measurement in clinical research: a review of methodology. Schizophr Bull.

[B74] Nyman H, Nybäck H, Wiesel F-A, Oxenstierna G, Schalling D (1986). Neuropsychological test performance, brain morphological measures and CSF monoamine metabolites in schizophrenic patients. Acta Psychiat Scand.

[B75] Jacobsen LK, Rapaport JL (1998). Research update: Childhood onset schizophrenia: implications of clinical and neurobiological research. J Child Psychol Psychiat.

[B76] Cabeza R, Nyberg L (2000). Imaging cognition II: an empirical review of 275 PET and fMRI studies. J Cogn Neurosci.

[B77] Kemali D, Maj M, Galderisi S, Ariano MG, Starace F (1990). Factors associated with increased noradrenaline levels in schizophrenic patients. Prog Neuropsychopharmacol Biol Psychiatry.

[B78] Van Kammen DP, Kelley ME, Yao JK, Gilbertson MW, Gurklis JA, Inosaka T, Saito H, Peters JL, Sato M (1996). Predicting haloperidol treatment response in chronic schizophrenia. Psychiat Res.

[B79] Di Rocco A, Bottiglieri T, Dorfman D, Werner P, Morrison C, Simpson D (2000). Decreased homovanillic acid in cerebrospinal fluid correlates with impaired neuropsychologic function in HIV-1-infected patients. Clin Neuropharmacol.

[B80] Amin F, Seeman TE, Mohs RC, Davidson M, Knott P, Berkman LF, Albert M, Blazer D (1994). Plasma homovanillic acid and performance on motor and cognitive tasks in community dwelling elderly. Neuropsychopharmacol.

[B81] Gilbertson MW, Yao JK, van Kammen DP (1994). Memory and plasma HVA changes in schizophrenia: are they episode markers?. Biol Psychiatry.

[B82] Medalia A, Gold J, Merriam A (1988). The effects of neuroleptics on neuropsychological test results of schizophrenics. Arch Clin Neuropsychol.

[B83] Oades RD (1985). The role of noradrenaline in tuning and dopamine in switching between signals in the CNS. Neurosci Biobehav Rev.

[B84] Brauns H, Haun D, Steinmann S (1997). The construction of an internationally comparable classification by class. (Erwerbsstatistische Besonderheiten am Beispiel von Labour Force Surveys der Bundesrepublik Deutschland, Frankreichs, Großbritanniens und Ungarns) Arbeitspapiere Arbeitsbereich 1/22.

